# The Dutch Citizen Forum on Public Reimbursement of Healthcare: A Qualitative Analysis of Opinion Change

**DOI:** 10.34172/ijhpm.2020.81

**Published:** 2020-06-15

**Authors:** Maarten Jansen, Rob Baltussen, Leon Bijlmakers, Marcia Tummers

**Affiliations:** Department for Health Evidence, Radboud Institute for Health Sciences, Radboud University Medical Center, Nijmegen, The Netherlands.

**Keywords:** The Netherlands, Public Deliberation, Citizen Forum, Priority Setting, Benefit Package Design

## Abstract

**Background:** A deliberative Citizen Forum ‘Choices in healthcare’ was held in the Netherlands to obtain insight into the criteria informed citizens would propose for the public reimbursement of healthcare. During 3 weekends, 24 citizens participated in evidence-informed deliberation on the basis of 8 case studies. The aim of this study was to assess how the opinions of 8 participants in the deliberative Citizens Forum changed and if so, why participants themselves believe their opinions have changed, whether participation influenced their perceived reasonableness of other participants in the forum and whether it influenced their opinions about involvement of citizens in decision-making.

**Methods:** Semi-structured interviews were held with 8 participants before and after their participation in the Citizen Forum. Using the method of reconstructing interpretive frames opinions about the public reimbursement of healthcare were reconstructed.

**Results:** Participants’ opinions changed over time; they became more aware of the complexity of decision-making and came to accept that there are limits to the available resources and accept cost as a criterion for reimbursement decisionmaking. Participants report that exchanging arguments and personal experiences with other participants made them change their initial opinions. Participants ascribed increases in the perceived reasonableness of other participants’ opinions to feelings of group-bonding and becoming more familiar with each other’s personal circumstances. Participants further believe that citizens represent an additional opinion to that of other stakeholders and believe their opinions should be considered in relation to those of other stakeholders, given they are provided with opportunities for critical discussion.

**Conclusion:** Organized deliberation should allow for the exchange of arguments and the sharing of personal experiences which is linked to learning. On the one hand this is reflected in the uptake of new arguments and on the other hand in the revision, specification or expansion of personal argumentation. Providing opportunities for critical deliberation is key to prevent citizens from adhering to initial emotional reactions that remain unchallenged and which may no longer be supported after deliberation.

## Background

**Key Messages**

**Implications for policy makers**

- Policy-makers who wish to involve uninformed citizens in benefit package design should allow for deliberation – we show this influences participants’ opinions about the reimbursement of healthcare.
- Such deliberation should allow the exchange of arguments and sharing of personal experiences – we show this is linked to learning.
- Such deliberation should allow opportunities for critical discussion – we show this is key for involvement.

**Implications for the public**

The outcomes of this study demonstrate that uninformed citizens are both willing and able to participate in difficult discussions about the public reimbursement of healthcare. If uniformed citizens are provided opportunities to deliberate, they learn from each other and are prepared to revise their first reactions which may be mainly guided by emotions. This learning is reflected in both the uptake of new arguments and the revision, specification or expansion of personal argumentation. Organized deliberation should allow for the exchange of arguments and the sharing of personal experiences.

In the Netherlands, the minister of Health, Welfare and Sport decides about the reimbursement of health services and their subsequent uptake into the Dutch national health insurance scheme. The Dutch National Health Care Institute (ZiNL) advises the minister by assessing health services on 4 criteria (effectiveness, cost-effectiveness, necessity, feasibility) and further appraises the health service on other identified criteria throughout their recommendation process.^1^ Stakeholders, including citizens, are provided opportunities to inform this process by sharing their views during public appraisal meetings^1^ although citizens are generally not present. Nevertheless, negative reimbursement recommendations by ZiNL, or final decisions by the minister, frequently attract public criticism. At the same time, the Dutch government is being confronted with policy-makers and scholars who caution them against budget increases in healthcare that cannot be sustained.^2^

The potential value of organizing public deliberation to achieve a better alignment between health policy decisions and informed perspectives from the public is increasingly receiving attention.^3-11^ Providing the public with opportunities for deliberation to inform policy-making is considered more legitimate as this can make policies more responsive to public values and can increase its transparency.^5,12^ Moreover, previous studies show that providing opportunities for deliberation tends to influence opinions to some extent,^4,13-28^ suggesting participants’ initial opinions on complex topics like health care are open to change and may differ from participants’ informed opinions.

It has long been argued that deliberative methods may improve the quality of uninformed opinions on political and social issues.^29^ Through practical reasoning participants deepen their understanding of their own preferences and those of others.^26^ They may replace uninformed opinions by views that are more rational, better supported by arguments and perhaps more consistent with their overall ‘belief system.’^30^ Opinions may become ‘enlightened’ as a result of deliberation, reflected by pre-deliberative opinions being ‘updated’ or revised based on ‘new information,’ which can be anything from facts to arguments.^27^ In the end, both the direction and the strength of individual opinions may change, albeit selectively depending on the quality and diversity of the information (or communication) as well as participants’ willingness to keep an open mind.^25-27^ Moreover, engaging in democratic deliberation may improve the mutual understanding among participants,^26^ or in other words the ‘political tolerance’ of other participants’ opinions or their ‘perceived reasonableness.’

This does not mean that opinion change necessarily results from reasoned processes of deliberation.^25^ Opinion change may result from harmful group dynamics, eg, if participants lack equal voice they may assimilate to the opinion of more effective participants.^25^ On the other hand, knowledge and attitudes developed before taking part in deliberations may decrease people’s willingness to consider diverging information and arguments, resulting in reluctance to opinion change.^25^

Multiple deliberative methods have been developed that can be used to elicit informed public opinions while taking into account well-known aspects of group dynamics, such as group dominance, groupthink and addressing unequal power. These methods differ in their structure, the public involved and their link to actual policy processes.^3,11^ Minimal requirements for the robustness and reliability of deliberative processes have been proposed and include (*i*) the provision of balanced factual information to participants, (*ii*) the inclusion of a diverse range of potentially conflicting perspectives, including minority and marginal perspectives, and (*iii*) the creation of opportunities for open discussion among participants to challenge and test competing claims.^31^

During 3 weekends in the fall of 2017 (October–November), the deliberative Citizen Forum ‘Choices in healthcare’ was held in the Netherlands. The objective of the Citizen Forum was to obtain insight into citizens’ informed preferences and identify criteria they would propose for decisions pertaining to the composition of the benefits package.^32,33^ The aim of this qualitative study was to provide insight into how the opinions of individual participants in a deliberative citizens forum change and if so, why participants themselves believe their opinions have changed. In addition, this study aimed to provide insight into whether participation influenced their perceived reasonableness of other participants, and their opinions about the involvement of citizens in healthcare decision-making. With this study we further intend to contribute evidence to the existing literature on opinion change and the potential of applying deliberative methods to inform choices in healthcare.

## Methods

###  Eligibility and Recruitment of Participants

Participants in the Citizen Forum were selected from an existing panel compiled by *Motivaction*, a research and consultancy agency specialized in the relations between values, motives, lifestyle and behavior.Participants were recruited for diversity at 2 aspects, to ensure as much as possible the representation of diverse views held by Dutch citizens.^34^ The first aspect of diversity relates to participants’ mentality groups (ie, attitudes to life) that represent shared aspirations regarding work, leisure and politics, and show similar lifestyle and consumption patterns. The segmentation into 8 mentality groups is based on value orientation (eg, traditional, modern, postmodern) and status seeking (ie, low, middle, high).^35^ Three participants were selected from each mentality group (n = 24). The second aspect of diversity relates to socio-demographic characteristics, ensuring overall diverse recruitment according to age (18+), gender, geographical location and education level. Initial recruitment for participation in the Citizen Forum was done through an email invitation, which only indicated that the topic of deliberation would concern a social issue. Subsequently, for the interview study, one respondent from each mentality group was randomly selected to be interviewed (n = 8), both before and after their participation in the forum. Each pre-selected participant for this study was subsequently invited face-to-face during an introductory meeting about the forum, 2 weeks prior to the first weekend of the forum, and all agreed – some afterwards by email having requested some time to decide. Participants in the forum received a financial incentive for their participation (a flat fee), as well as free accommodation (2 nights during each of the 3 weekends) and free meals. Those who were interviewed received an additional financial incentive (also a flat fee).

###  The Citizen Forum – Topic and Structure of Deliberative Sessions

Two moderators guided deliberations on 8 selected case studies, which included preventive, diagnostic and curative services for a range of conditions: dental (orthodontic) braces for youngsters, Alzheimer’s disease, heart burn (pyrosis), attention-deficit hyperactivity disorder among children, atypical hemolytic uremic syndrome (a rare disease), total body scan, obesity, and hip prosthesis for elderly people. Deliberations took place in both small groups and plenary sessions. Participants received descriptions of the clinical manifestations and treatment options for each of the case studies. This information was validated by experts and presented in a neutral manner to avoid any bias where possible. The deliberations around each case typically lasted 2 to 3 hours, culminating in a listing of arguments in favor and against public reimbursement by participants. Participants further interacted in 3 separate sessions with experts: an ethicist, a health economist, and a specialist in health technology appraisal who was also a former member of ZiNL’s appraisal committee that advises the Minister of Health about reimbursement decisions. These interactions, solely based on questions put forward by the participants themselves, served to share personal experiences and deepen their understanding of dilemmas. A detailed agenda of the 3 weekends is listed in Supplementary file 1. More detailed descriptions of the methods, case studies and results of the Citizen Forum are presented elsewhere.^32,33^

###  Data Collection: Semi-structured Interviews 

The semi-structured in-depth interviews were guided by a predesigned topic guide (see Supplementary file 2). Interviews started with a short introduction by the interviewer explaining the context and goals of the interview. The interviewer then continued by inquiring about the overall meaning of the Dutch basic benefits package to the person interviewed, followed by questions identifying all arguments they believe should be used to decide whether specific healthcare should or should not be publicly reimbursed. Finally, respondents were asked what they think about the involvement of citizens in healthcare decision-making. For interviews after the Citizen Forum the same topic guide was used with the addition of questions asking them to reflect on any moments throughout the forum during which they felt their own opinions somehow changed and in what way. Furthermore, they were asked whether their perceived reasonableness of other participants’ opinions changed during their participation in the forum. Interviews were pilot tested with a second researcher (MT).

 The first round of interviews was conducted between the introductory meeting of the forum and its first weekend. The second round of interviews took place in the weeks following the final weekend. Interviews took place at the interviewees’ homes and were conducted by one of the authors (MJ). Participants signed informed consent forms. Interviews were audio recorded and lasted between 45 and 120 minutes. In 4 out of 16 interviews, one of the interviewee’s relatives was present in the same room during the interview, but none of them participated or intervened in the interview process. The interviewer made notes and wrote down important observations directly after the interviews.

###  Data Analysis: Reconstructing Interpretive Frames

Interviews were transcribed verbatim after which interpretative frames were reconstructed for each theme addressed by the participants. The term ‘interpretive frame’ refers to a quadruple set of elements that together comprise a respondent’s view about a topic, in this case concerning the desirability of using certain criteria in healthcare decision-making: (*i*) a person’s context-specific problem definition, (*ii*) perceived solutions, (*iii*) empirical and ethical background theories, and (*iv*) normative preferences.^36^ Transcripts were coded, making a distinction between these 4 layers of interpretive frames. For each transcript 2 researchers (MJ, MT) independently reconstructed the interpretive frames of that interviewee in Microsoft Word. The resulting frames for each of the interviewees were compared and any differences discussed until settled between the researchers, resulting in revision of frames if necessary. Finally, interpretive frames of interviewees were combined together for each of the themes, while still differentiating between views held before and after. Individual contributions to summarized theme narratives are numbered 1–8, each corresponding to a particular participant, which allows insight into individual-level opinion changes that would be concealed in the aggregated before/after comparisons that reflect net opinion changes only. See Table for an overview of participant characteristics. Finally, summaries of change were constructed for each theme reflecting the overall observed changes in opinion. Replies to questions asking participants what they think about the involvement of citizens in decision-making and the additional questions asked only after the forum were extracted from interview transcripts, combined and summarized as narratives for each question in Microsoft Word. Illustrative quotes are added in *italics*.

**Table:** Characteristics of Interviewed Participants (n = 8)

**Participant ID**	**Age**	**Gender (Male/Female)**	**Geographical Location**	**Highest Education Attained**	**Mentality**
1	30	Female	West	University	Social climber
2	24	Male	Middle	College	Cosmopolitan
3	40	Male	North	Vocational training	Traditional
4	70	Male	South	College	Modern mainstream
5	30	Female	South	Vocational training	Convenience-oriented
6	50	Female	Middle	College	New conservative
7	32	Female	West	College	Post-materialist
8	28	Female	South	University	Post-modern hedonist

## Results

Firstly, we present an overview of participants’ characteristics in Table. Secondly, based on the analysis we identified 6 themes: *costs and medical necessity, evidence of effectiveness, personal responsibility, lifestyle and prevention, age, financial barriers* and the* number of patients in need*. For each theme, we present a short summary of the observed changes in participants’ opinions, followed by a more detailed description of their opinions *before* and *after* deliberation. Thirdly, self-reported reasons for changes in opinions and changes in the perceived reasonableness of other participants’ opinions are presented. Finally, participants’ opinions about the involvement of citizens in decision-making are presented.

###  Narrative Summaries of 6 Identified Themes

####  1. Costs and Medical Necessity

*Summary of change:* Participants initially defined their problem as that the government does not reimburse all basic medical care. Afterwards, participants redefined the problem as that the government is not able to reimburse all basic medical care because of a limited health budget. It became acceptable to consider the financial feasibility of reimbursements.

*Before: *Several participants find it problematic that not all basic medical care is reimbursed (ID 1, 3, 4, 5, 7, 8). They define basic medical care as health services that are needed for ‘illnesses or conditions that simply happen to a person’ and help people to restore their functioning or contribute to the quality of their lives. Participants are of the opinion that the government bases its decisions disproportionately on costs and insufficiently on quality of life considerations (ID 1, 4, 5, 7). Politicians prefer to reimburse luxury care, such as cosmetic treatments, instead of medically necessary care, (ID 3, 4, 5, 7) and appear unaware of the basic needs of the population (ID 3, 4). Some participants believe that the available healthcare budget is sufficient to fund all basic medical care and, if not, that the budget can be increased by lowering other government budgets. They consider health as the number one priority (ID 1, 5, 7) and find it inappropriate to consider costs if a patient’s life is at risk (ID 5, 8).

*After:* All 8 participants find it problematic that not all basic medical care can be reimbursed within the healthcare budget, even when healthcare budgets are increased in the future (ID 1, 5, 8). One participant felt that the quality of care is at risk if not the right reimbursement choices are made under budget constraints (ID 7). Simple and clear limits should be set to which care is reimbursed and which care is not (ID 7). Most participants state that health services for life threatening conditions should always be reimbursed (ID 2, 3, 5, 6, 8) and costs should in principle not preclude health, though some believe that the best decisions are made by considering both medical necessity and financial feasibility (ID 1, 2, 5). In line with this reasoning, they consider calculating the cost per quality adjusted life year (cost/QALY [quality-adjusted life year]) an appropriate tool for making reimbursement decisions, with a higher medical necessity justifying a higher cost/QALY (ID 2, 5). They do not consider cosmetic treatments as medically necessary care (ID 1, 2, 3, 6, 7) and one participant believed costs can be significantly reduced if the government is more strict in reimbursing cosmetic treatments (ID 7). Some believe the minister should negotiate drug prices with pharmaceutical companies (ID 4, 6), preferably in cooperation with other European Union member states (ID 4) and reject reimbursement in case drugs are too expensive (ID 6). Ideally the prices of health services are strongly restricted (ID 5).

“*Choices have to be made, unfortunately, otherwise you run short of funding for other forms of care. Before participating I always used to say “you should help everyone”, and I still believe we should, but sometimes you just can’t, because there is not enough money to do so” *(ID 5).

####  2. Evidence of Effectiveness 

*Summary of change:* New arguments in favor of requiring evidence of effectiveness emerged, namely: the potential to save costs by not reimbursing ineffective care and protecting people from being provided with false hope. Claims of doctors and patients that a health service works were considered sufficient to meet this demand for evidence.

*Before:* Some participants believe health services qualify for public reimbursement if there is evidence of effectiveness (ID 1, 5) or a visible and measurable effect (ID 3). If evidence of effectiveness is lacking and the condition is not life threatening the health service should not be reimbursed so that money can be spent on services that are proven to be effective or lifesaving (ID 3, 5). Health services for life threatening conditions should always be reimbursed regardless of evidence (ID 3, 5, 8). Reimbursing expensive health services with limited effects is considered a waste of public funding (ID 3, 4, 7). One participant claims scientific evidence of effectiveness is not necessary to justify reimbursement: it would be sufficient if patients report they benefit from a health service and if most citizens have positive attitudes towards its use (ID 7).

*After:* Four participants state that in addition to evidence of effectiveness, the claims of doctors or patients that a particular health service might work already justifies reimbursement (ID 1, 2, 5, 8), with one participant suggesting that one should do everything possible to improve someone’s health (ID 8). Treatments for life threatening conditions should always be reimbursed and if a condition is not life threatening then there should be at least ‘50% evidence of effectiveness’ (ID 5). Another holds the opinion that at least a visible and measurable effect should be present (ID 3). One participant explains that requiring evidence of effectiveness protects people from being provided with false hope in ineffective treatments (ID 6). One participant became explicit about the desirability of not reimbursing technologies that lack evidence of effectiveness to save costs (ID 7).

*“If 2 treatments for the same condition can potentially be reimbursed, and a choice has to be made between them, scientific evidence should be decisive. However, due to personal stories from other participants in the forum, I now feel that whenever this is not the case it is also sufficient if doctors are of the opinion that it works” *(ID 1).

####  3. Personal Responsibility, Lifestyle and Prevention

*Summary of change:* Several participants proposed that lifestyle support programs should be reimbursed to provide patients a chance to show they can take responsibility for their own health, while explicitly adding that people should remain free to make their own choices in life and should not be belittled.

*Before:* Participants claim health services should not be reimbursed if their need is the result of taking calculated risks (ID 1, 2, 3, 5) or immoral behavior (ID 4). Making people aware of their lifestyle should be promoted by means of reimbursing prevention programmes, which is believed to be cost saving (ID 4) and able to prevent worse long-term outcomes (ID 8). The continuation of reimbursement of certain health services should become dependent on the patients willingness to make lifestyle changes (ID 4). Certain participants were cautious in restricting reimbursement on the basis of personal responsibility, as there are usually underlying causes for specific lifestyles (ID 5, 7, 8). In addition, 2 participants believed some citizens are simply not capable of taking their responsibility, thereby justifying the reimbursement of programs that support them in taking responsibility (ID 6, 7).

*After:* Some participants claimed health services should not be reimbursed if their need is a result of individuals taking calculated health-related risks (ID 1, 3, 4). Those taking calculated risks may seek other funding opportunities such as crowdfunding or payment in terms (ID 3). One participant believed prevention programs should be reimbursed if there is evidence that they are cost-saving (ID 4). Two participants claimed a patient must take his/her personal responsibility to improve their respective condition before the needed technology will be reimbursed (ID 2, 8) – although adding technologies should always be reimbursed in case of medical necessity (ID 2). Others instead claimed support programs to help patients change their lifestyle should be reimbursed to give people a chance to show they can actually take their responsibility (ID 4, 5, 6, 7, 8). However, ideally patients pay for healthcare themselves if a technology is needed as a result of their own behavior (ID 6). Two participants believed there are always underlying causes, explaining someone’s behavior and stressed we should be cautious to judge these people (ID 5, 8). Also, people should not be belittled and remain free to make their own choices (ID 5). One participant added that the use of personal responsibility in decision-making is only warranted as a last resort under budget constraints (ID 8).

####  4. Age

*Summary of change:* The argumentation against using age as a decision criterium was further specified afterwards by explicitly linking it to questions of equal access, the appropriateness of using age as a prediction factor and questioning the discriminatory nature of cost-effectiveness. Arguments in favour of using age emerged too, claiming there is relatively less benefit gained at higher costs when treating older people compared to younger people.

*Before:* Three participants consider the use of age in reimbursement decision-making as discrimination or a violation of equality (ID 1, 6, 8), even in the case of a limited budget (ID 1). One participant points out that treatment at old age should not automatically be reimbursed because the chance of success of treatment is dependent on age (ID 4).

*After:* Participants believe personal characteristics should not be part of the decision criteria (ID 1, 2, 5, 8). Everyone should have equal access to care (ID 2) and as someone’s life may end anytime, age is not useful as a predictive factor (ID 5). One participant claimed the use of methods that discriminate against elderly people, like the cost-per-QALY method, is unjustified on this basis (ID 1). Another claimed only increased risks associated with the use of health services at an older age justify specifically not reimbursing health services at an older age (ID 7). According to one participant, the more restrictive reimbursement of health services for older people can be justified by the need to reduce total healthcare costs because (*i*) care for elderly people is more expensive, (*ii*) elderly people in general enjoy a lower quality of life and (*iii*) they are less likely to benefit long-term from treatments (ID 3). If a choice must be made between younger and older people, younger people should have priority (ID 4).

####  5. Financial Barriers

*Summary of change:* Paying out-of-pocket for treatments became acceptable for treatments that are relatively cheap and at the same time used by many, provided that high costs at the individual level due to adding up of costs are prevented. Special concern for those with the lowest incomes shifted towards concern for those with incomes just above modal, for whom it was believed support is lacking the most.

*Before:* Out-of-pocket costs for healthcare are perceived as extremely high (ID 6). Two participants argue it is unacceptable that costs prevent people from timely seeking care (ID 1, 5) – which in turn negatively affects the economy (ID 1). One participant stressed the need to remove financial barriers to visit the general practitioner, arguing it is important to discover diseases as early as possible (ID 3). Two participants stressed the need for solidarity and ensuring accessibility of care for those who are unable to pay out-of-pocket (ID 7, 8). Several participants believed it would be fair to make richer citizens pay relatively more compared to poorer citizens to abolish costs at the point of care (ID 5, 6, 8) with one claiming it would be preferable to have everyone pay a bit more (ID 1). On the other hand, one participant argued out-of-pocket contributions are sometimes preferable as this motivates people to carefully balance if they need the technology in the first place (ID 4).

*After: *Four participants believed that if a treatment is cheap, used by many patients and costs are acceptable to pay out-of-pocket, the treatment should not be reimbursed (ID 5, 6, 7, 8), with the caveat that it should not result in high costs at the individual level due to adding up of costs of different treatments (ID 5, 6). Three participants stressed the benefits package should be based on solidarity and equality and that this implies that costs are reimbursed for those unable to pay out-of-pocket (ID 1, 3, 4). It is further believed that people with an income just above modal experience the largest consequences of out-of-pocket payments, while for those who are poor there is all sorts of support available (ID 4). Especially financial barriers to visiting a general practitioner and hospital care should be removed (ID 4).

####  6. Number of Patients in Need 

*Summary of change:* Before, participants argued that only frequently used healthcare should be reimbursed, or otherwise paid for via crowd-funding, but after the Citizen Forum it is considered inappropriateness to burden people with the task of raising funds themselves and crowd-funding being considered a too slow mechanism for doing so.

*Before:* Participants believed that if the government has to make choices in healthcare only those health services should be reimbursed that are frequently used (ID 2, 7). Although not providing less frequently used health services is problematic in case citizens are not able to manage their health state by themselves (ID 7). For (other) technologies that only a few people use, including expensive orphan-drugs, crowd-funding can be used to support financial access (ID 2, 7). If someone is unsuccessful in raising private funds it is argued one should learn to accept this (ID 7).

*After:* One participant now specifically considered it incorrect to only reimburse health services if they are frequently used on the basis of 2 arguments: (*i*) it is inappropriate to burden people with the responsibility of raising funds to improve their quality of life, and (*ii*) crowd-funding is to slow as a mechanism to raise funds (ID 2). Nevertheless, another participant mentioned that the basic benefits package is there in principle to reimburse care that is often used (ID 5). As we as a community pay for the reimbursement of care, we should all benefit from it (ID 5). Infrequently used care, such as expensive treatments for orphan diseases, would ideally be reimbursed but it is not preferable to reimburse all treatments in this category at the cost of more frequently used care (ID 5, 8).

*“… should you then say, because only few people use a specific health service, that it should not be included in the benefits package and people should just take care of funding themselves?” *(ID 2).

###  Self-reported Reasons for Changes in Opinions

 Participants claim their opinions changed after participating in the Citizen Forum, albeit in varying degrees. Afterwards, participants report that they have become more aware of the complexity of making reimbursement decisions; for every argument in favor of public reimbursement an argument against can be found. Public reimbursement has become less black or white. Three participants describe their overall change in opinions as achieving a better balance between more ‘intuitive emotional responses’ and more ‘rational responses’ (ID 5, 6, 8).

*“Yes, you can’t help everyone unfortunately. That is my ratio speaking, but my feelings tell me ‘you should help everyone.’ That is the biggest change. I still believe you should help everyone but that is not realistic” (*ID 5).

 Several participants claim their opinions changed due to the continuous engagement in discussions about concrete cases and critically questioning each other’s arguments (ID 2, 3, 4, 5, 6, 7, 8), or being confronted with new arguments (ID 3, 4). One expressed the feeling that being provided with more time to deliberate would result in even further changes in opinions (ID 8).

*“The detailed discussions during the citizen forum make that you become better able to weigh things and that you can better motivate your choices” (*ID 8).

*“The arguments of participants have contributed. […] Just like during one specific case when someone said ‘I’m not doing it’ which makes you think and become aware of the other side of the discussion (ID 4).*”

 For some participants the weight of certain arguments changed after being confronted with personal experiences of other participants (ID 1, 2, 7).

 Finally, some participants claim the interaction with experts has contributed to their change (ID 1, 2, 5, 6, 8). Their contribution is linked most clearly to self-reported changes in reasoning about the role of costs and financial sustainability in relation to reimbursement decision-making (ID 1, 2, 5, 6, 8). Expert knowledge is perceived as trustworthy.

###  Self-reported Changes in the Perceived Reasonableness of Other Participants’ Opinions

 Participants unanimously report to perceive certain opinions held by other participants as relatively more reasonable after their participation in the Citizen Forum.

*“…I also find myself thinking “OK, if you look at it from that perspective I can understand where you are coming from”. Even though I did not agree with everything, but you do get a better appreciation of each other’s arguments” *(ID 7).

 Some participants explicitly mention this increase in perceived reasonableness does not apply to what participants describe as ‘extreme opinions’ (ID 2, 3, 5, 7, 8).

*“… one person was always against reimbursement in a very stubborn way and then I thought to myself “Gosh, that person just doesn’t think any more than he has to” *(ID 7).

 Finally, some participants ascribe part of this increase in perceived reasonableness of other participants’ opinions to becoming familiar with the personal life stories of other participants (ID 2, 4, 6) and experienced group-bonding during the weekends (ID 2).

*“In general I did come to perceive other people’s arguments as more reasonable […]. I believe this is the result of getting to know people better, and knowing their backgrounds. And, because you bond with people over time” *(ID 2).

###  Changes in Opinions of Participants About the Involvement of Citizens in Decision-Making

 Before participation in the Citizen Forum participants claimed medical doctors should be the ones to decide on the reimbursement of health services (ID 1, 3, 4, 5, 6). They have medical knowledge (ID 1, 3, 4, 6) and know what patients can afford (ID 5), which is considered of more importance than the financial perspective used by the government (ID 1, 3, 4, 5, 6). Ideally, reimbursement decisions should result from cooperation between politicians, doctors, and other interest groups, such as patients and citizens (ID 2, 3, 4, 6, 7). Overall, the government should listen more carefully to its citizens and better involve them in decisions (ID 2, 3, 5, 7), and that results of involvement should be used (ID 3, 4, 5, 8). On the other hand, 2 participants further felt that citizens in general do not necessarily represent the collective interest and put their own interests first (ID 1, 2), while adding medical doctors could best represent the collective perspective (ID 1). In addition, one participant raised concerns about whether medical doctors can also represent the perspective of healthy citizens (ID 6). Participants proposed to involve citizens as critical assessors of decisions (ID 2, 6), or to organize participation in the form of a representative Citizen Forum (ID 4, 6, 7). In addition, 2 participants considered a survey an inappropriate method to explore the opinions of citizens (ID 4, 8) as it is thought to have no impact on decision-making (ID 4). One participant claimed it is currently unclear how reimbursement decisions are made in the first place (ID 8).

 Afterwards, participants express the opinion that conscious decisions require the cooperation among politicians, as representatives of the public (ID 1, 2, 6), economists, who can decide on the financial feasibility (ID 1) and doctors, who have the knowledge to determine whether health services are basic medical care (ID 1, 2, 6). Others believe politicians (ID 4) or experts (ID 5, 7, 8) should take these decisions. With regard to involving citizens certain participants continued to express the opinion that citizens should be involved in decision-making (ID 3, 5, 7), while another participant now framed this as a democratic right (ID 8). On the other hand, one participant continued to believe medical doctors are best able to represent citizens’ interests from a medical perspective and better able to judge the medical necessity of treatments from a collective perspective, explicitly arguing against citizen participation in decision-making (ID 1). It was further considered necessary by participants to communicate in which way their arguments have been considered in decision-making to improve the legitimacy of reimbursement decisions (ID 2, 3, 4, 6, 8). Translating decisions to citizens requires explanations about what is and what is not financially feasible (ID 1, 4). One participant afterwards believed one-sided reporting by the popular media should be addressed, as they fail to represent the argument that reimbursing one health service comes at the cost of no longer being able to reimburse other services (ID 4). Moreover, participants believe that the opinions of individual citizens should never be decisive in itself, rather, their arguments should be considered in relation to those of other stakeholders (ID 2, 3, 5, 8). Afterwards, it was argued that online surveys are considered insufficient as they lack critical discussion (ID 2, 4, 5, 8).

## Discussion

 In this study we assessed how deliberation influenced participants’ opinions about choices regarding the composition of the benefits package by comparing their interpretive frames before and after the Citizen Forum. Overall, participants became more aware of the complexity of decision-making and accordingly revised their opinions on how reimbursement choices should be made. Participants came to accept that there are limits to the available resources – in particular health budgets – and accept a role for costs despite several participants’ initial opinion that this is unacceptable. Participants report changes in their opinions are the result of exchanging arguments with other participants and sharing personal experiences. Increases in the perceived reasonableness of other participants’ opinions are ascribed to feelings of group-bonding and becoming more familiar with each other’s personal circumstances. Participants further believed that citizens represent an additional opinion to that of other stakeholders and believe their opinions should be considered in relation to those of other stakeholders, given they are provided with opportunities for critical discussion. An opportunity to deliberate is considered a must for valuable contribution, guaranteeing that citizens not just represent their first emotional reaction.

 The opinion change of individual participants was caused by new arguments that they had included (eg, new evidence of effectiveness), the revision of (strong) prior opinions (eg, on costs or medical necessity; or number of patients in need) and the specification or expansion of argumentation (eg, on financial barriers or personal responsibility; lifestyle and prevention). For certain topics opinion change went in opposite directions (eg, evidence of effectiveness) while for other topics the deliberation resulted in increased convergence of opinions (eg, cost and medical necessity). Overall, reframing of the problem for most topics came with a change in aggregated preferences for how these topics would need be taken into consideration in healthcare reimbursement decision-making.

To our knowledge, this study is the first in the Netherlands to over time and qualitatively examine changes in citizens’ opinions towards the public reimbursement of health services when provided ample opportunity for deliberation. A second study, performed in parallel during the Citizen Forum, used Q-methodology to assess participants’ opinions in a quantitative way.^37^ In this exercise participants were asked both before and after the citizens forum to rank-order a set of short written statements about the reallocation of healthcare, using a range from most agreeable to least agreeable. The study showed that: (*i*) participants’ support for prioritization in healthcare generally increased after participating; (*ii*) participants became more considerate of healthcare costs and (*iii*) cost-effectiveness emerged as a relevant criterion for setting priorities in healthcare. These results are in accordance with the results reported in this paper.

Previous studies using deliberative methods to gather input from citizens on healthcare related issues have similarly shown that deliberation tends to influence people’s opinions.^4,13-22^ Due to much heterogeneity in context and the study methods used, a systematic comparison of the results of these studies is not feasible. Nevertheless, several previous studies do demonstrate that deliberation influences participants’ acceptance of choices in healthcare under budget constraints.^19-21^ In one specific study, aimed to test the assumption that the public will reject any explicit consideration of costs in coverage policy, the authors conclude that participants who understood cost-effectiveness analysis, were largely open to its use, and changed their own funding priorities when given cost-effectiveness ratio information.^22^ Another study concludes that when working with a limited budget, participants supported the exclusion of high-cost, low-value interventions, among others.^38^ Taken together with results from this study, this suggests that through deliberation participants may come to accept a role for costs and affordability considerations in decision-making.

Although we did not explicitly test for factual knowledge gains by participants, which would be an indication that opinion changes are the result of individual learning and reflection (rather than merely of some social group dynamic), it does appear that participants have become more aware of the complexity of decision-making and the broader decision-making context. Self-reported reasons for changes in opinions demonstrate that participants engage with and reflect on what is being said by others and how this information relates to their own opinions. This reflection is argued to be a critical dimension of what Himmelroos et al refer to as ‘deliberative reasoning.’^25^ Other dimensions of deliberative reasoning include the referring by participants to the common good, and their refraining from being disrespectful towards other participants.^25^ Participants did not explicitly use the term ‘common good.’ They did refrain from being disrespectful towards other participants and, in fact, at least some participants expressed empathy with the personal life stories of other participants and hence understanding of the opinions they expressed. Both reflection and refraining from being disrespectful towards other participants are known to be strongly associated with the willingness of participants to change their opinions,^25^ which may have contributed to the opinion change observed in this study.

Three participants of the Citizen Forum (ID 5, 6, 8) reflected on changes in their opinions as achieving a better balance between more ‘intuitive emotional responses’ and more ‘rational responses,’ with one participant feeling better able to motivate decisions not to reimburse a technology in spite of feeling intuitively guilty at first. This disjunction is recognized in Kahnemans ‘theory of thinking fast, thinking slow’ which describes 2 modes of thought: “System 1” is fast, instinctive and emotional; “System 2” is slower, more deliberative, and more logical – with system 1 thinking often being associated with biased views.^39^ It can be argued that deliberation caused participants to move away from their more frequently biased system 1 thinking towards more reasoned system 2 thinking. This raises concerns about the validity of citizens’ responses on health policy decision in case they are not provided with sufficient opportunities to deliberate. Further illustrating this point, participants came to believe surveys are not fit-for-purpose to inform decision-making. Surveys are believed to lack the necessary background information and critical discussion that is perceived as crucial by participants to prevent citizens from supporting positions they actually would no longer want to support after deliberation. Dolan et al mentioned that public’s views about setting priorities in healthcare are systematically different when they have been given an opportunity to discuss the issues. Surveys that do not allow respondents time or opportunity for reflection may be of doubtful value.^15^ In a similar vein, Maxwell et al conclude that engagement of the public is more costly than polling but essential when opinions are unstable and difficult decisions must be made.^14^ Therefore, if health authorities seek to inform their decision-making process with citizens’ opinions as part of efforts to legitimize their decision-making, it seems warranted they do so by providing citizens with deliberative opportunities.

 Our study has several strengths and limitations. The strength of this study is that it comprehensively captures how deliberation influences participants’ interpretive frames and thereby is able to illustrate the diverse changes that occur due to deliberation. This differs from more quantitative approaches that try to capture changes in opinions, eg, using Q-methodology. Another strength is the representation of diverse perspectives in this study by selecting participants from 8 different mentality groups. Although the interviewees were relatively young on average, this study shows that citizens with different backgrounds do reconsider their opinions about choices in healthcare reimbursement. At the same time, the sample size of 8 participants is a limitation in itself. Inclusion of more or other participants from the Citizen Forum might have resulted in different results, although the aim of this study was not to represent society at large but to provide insights into how opinions of participants in the Citizens Forum had changed and what had made them change their opinion. We believe the processes that took place are likely to be generalizable to larger groups of citizens if they participated in similar deliberative exercises. Furthermore, the preselection of participants in the Citizen Forum may have resulted in overrepresentation of participants who are highly motivated to engage in deliberations and more open to opinion change. This is in fact a limitation of any form of organized deliberation. One further limitation is that summaries of reconstructed interpretive frames were not sent to participants for feedback before and after the Citizen Forum. Although this type of respondent validation is valuable, we feared that participants would end up using these summaries as a personal reference point for staying consistent over time during the Citizen Forum. If used this way, summaries could become a barrier to the learning process the Citizen Forum was designed to facilitate. Another limitation is that participants were confronted with reallocation statements as part of the Q-methodology exercise before their first interview was conducted which may have already influenced their opinions on certain themes. Furthermore, the impact of experts on the quality of participants’ argumentation is influenced by and dependent on how much time participants were provided to ask questions to experts, who could challenge and potentially improve the quality of their argumentation. In addition, the selection of specific experts themselves may have influenced the views of participants, although experts were instructed to refrain from presenting their personal opinions in response to questions by participants and to present balanced views in their field of expertise to the best of their ability. Also, the observed learning curve may not just be the result of participation in the Citizen Forum. It is likely that participants learned, or were influenced, by factors outside the Citizen Forum that they were unable to recollect during interviews, eg, exposure to news coverage, deliberations with their peers or their own private search for information about the subject. However, it could be argued this is part of daily life and therefore inherent to the application of deliberative methods that organize multiple meetings over time.

## Conclusion

 The results of this study show that actively involving citizens in a deliberative Citizen Forum contributes to their revaluation of the complexity of decision-making and the need to make difficult choices, while becoming more receptive to costs as a criterion in healthcare decision-making. Participants believe that citizens represent an additional opinion to that of other stakeholders and should be involved in healthcare decision-making. Organized deliberation should allow for the exchange of arguments and the sharing of personal experiences. On the one hand this is reflected in the uptake of new arguments and on the other hand in the revision, specification or expansion of personal argumentation. Providing opportunities for critical deliberation is key to prevent citizens from adhering to initial emotional reactions that remain unchallenged and which may no longer be supported after deliberation.

## Acknowledgements

 The Netherlands Organisation for Scientific Research (NWO) funded this study (grant number 453-14-003). We would like to acknowledge Agnes Toll for transcribing the interviews.

## Ethical issues

 The Committee on Research Involving Human Subjects of the Radboud University Medical Center reviewed and waived ethical approval for this study (reference 2017-3444).

## Competing interests

 Authors declare that they have no competing interests.

## Authors’ contributions

 All authors contributed to the idea and design of this study. MJ performed the interviews after which MJ and MT independently analyzed the collected interview data. MJ and MT wrote a first version of the draft manuscript after which RB and LB gave feedback for revision.

## Supplementary files


Supplementary file 1. Programme of the Citizen Forum “Choices in Healthcare.”

Supplementary file 2. Interview Guide.
