# Supplementary material for: The Dutch Citizen Forum on Public Reimbursement of Healthcare: A Qualitative Analysis of Opinion Change
Source: Int J Health Policy Manag. 2020 Jun 15;11(2):118–27. doi: 10.34172/ijhpm.2020.81 (PMC9278612; doi:10.34172/ijhpm.2020.81)
Supplement: Supplementary file 1 — Programme of the Citizen Forum “Choices in Healthcare.” [file ijhpm-11-118-s001.pdf]

Supplementary file 1. Programme of the Citizen Forum “Choices in healthcare”

**First weekend**	
**Day 1**	Welcome, introductions, explanation of aim and working methods; announcement of house rules.
	Brief presentation of the Dutch healthcare system and the basic health insurance benefits package.
	Case study #1: Dental braces for children.<br>- 10 min introduction in plenary by moderator; viewing of a short explanatory film;<br>- participants working individually, indicating their preference in favour of or against inclusion of treatment in the benefits package and listing their arguments on post-its;<br>- opportunity to ask questions for clarification in plenary<br>- breakout sessions (4 groups; 6 pp per group) for small group deliberations;<br>- plenary: sharing of viewpoints and arguments in favour and against inclusion of treatment in the basic health insurance benefits package.<br>Similar procedure is used during case studies 2-8 (with the exception of case study 6, described below): participants working individually after introduction and explanatory film; breakout sessions in 4 groups (different composition); followed by plenary session for sharing of viewpoints and arguments.
	Case study #2: Alzheimer’s disease.
**Day 2**	Welcome, brief review of Day 1 proceedings.
	Case study #3: Heart burn.
	Case study #4: Treatment of atypical hemolytic uremic syndrome.

Second weekend	
**Day 3**	Review of Proceedings of 1st weekend; feedback from participants; expectations.
	Case study #5: Obesity.
	Participants questioning the expert in Ethics (plenary session; free interaction).
	Case study #6: Attention-deficit hyperactivity disorder among children.<br><br>Different procedure: after individual period for reflection and listing of arguments, participants chose to join one of 2 groups in the plenary session: proponents and opponents, facing each other and trying to convince each other.
**Day 4**	Participants questioning the expert in Economics (plenary session; free interaction)
	Case study #7: Hip prosthesis for elderly people.
	Case study #8: Total body scan.
**Third weekend**	
**Day 5**	Review of Proceedings of 2nd weekend; feedback from participants; expectations.
	Prioritising cases studies in breakout sessions: why include or exclude from basic health insurance?
	Free interaction with third expert: expert in health technology appraisal, former member of the ZiNL advisory committee (plenary session).
	Breakout session: appraising narrative text and quotes on identified inclusion/exclusion criteria.
	Breakout session: selecting elements for inclusion in the Manifesto.
	Breakout session: appraising narrative text and quotes on dilemmas.

Evening	Rapporteurs reviewing, systematising and selecting statements and quotes on inclusion/exclusion criteria and dilemmas – in preparation for the final day.
**Day 6**	Plenary session: final review of draft narrative text on inclusion/exclusion criteria and dilemmas.
	Individual pitches, formulating viewpoints to build 'argumentation clouds' (in plenary).
	Participants interviewing each other (in pairs; recorded by themselves on camera), addressing any of the following points:<br>- What was the most important insight that you have acquired?<br>- How serious have you taken your participation in the citizen forum?<br>- Did you feel free to express yourself?<br>What is your opinion about the general atmosphere during the citizen forum?
	Repeat of the individual Q-sort exercise.
	Oral process evaluation in plenary.
	Collective viewing of video recorded interviews.
	Next steps and Closure.
