# Supplementary material for: The Dutch Citizen Forum on Public Reimbursement of Healthcare: A Qualitative Analysis of Opinion Change
Source: Int J Health Policy Manag. 2020 Jun 15;11(2):118–27. doi: 10.34172/ijhpm.2020.81 (PMC9278612; doi:10.34172/ijhpm.2020.81)
Supplement: Supplementary file 2 — Interview Guide. [file ijhpm-11-118-s002.pdf]

Supplementary file 2. Interview Guide

Before the start of the interview:

- General introduction, explain the research goals, expected duration of the interview
- Informed consent, start the audio recording

Introduction:

- In the Netherlands, every citizen is obliged to have basic health insurance. This ensures citizens equal access to a so-called basic package of care. Not all the care that can potentially be delivered is in this basic package of care. Moreover, this package is not a single-time package, because new treatment options are constantly becoming available that can be added to the basic package, or for example, certain care included in the basic package may have become less relevant and the care may well be taken out of the basic package.

- **Objective:** We would like to ask you before the Citizens Forum about what you think are important considerations in determining whether or not care belongs in the basic package (or what you mean by valuable care) and would like to repeat this afterwards to see if your opinion on this has changed.

Questions:

- What does the basic package of care mean to you?

- What are your experiences with the basic package? What's going well/not well?

- Perhaps you know examples of care decisions that are not (anymore) reimbursed from your environment or the news? What do you think about this? What do such decisions mean to you?

- What do you understand to be valuable care?

    - What do you think are important considerations when determining whether or not care should be reimbursed through the basic package (whether care is valuable enough), or not?
    - What makes you think that these considerations should/should not play a role?
    - What is important to you?
    - Do you think the considerations you have mentioned will be well taken into account when the government decides? In your opinion, is there sufficient attention to such considerations?

- What do you think about making basic package decisions?
    - Do you think choices should be made at all?
        1. Should this choice lie with a practitioner, with policy-makers or politicians?
    - Do you have an idea of how decisions are made in practice?
    - How do you think the management of the basic package could be improved?
    - Do you feel that you are involved/heard in decisions about the basic package?
        1. How involved do you feel in decisions about the basic package?
        2. Do you need to be better involved in decisions?
        3. What would make you feel better involved in decisions or what could increase your support for decisions?

Additional questions after the Citizen Forum:

- Could you reflect on whether you can recall any moments during which your opinion somehow changed and in what way?
- Could you reflect on whether your perceived reasonableness of other participants has changed over the course of your participation in the Citizen Forum?

End: Do you have anything else you would like to share?
